# Effect of augmented nutrient composition and fertigation system on biomass yield and cannabinoid content of medicinal cannabis (*Cannabis sativa* L.) cultivation

**DOI:** 10.3389/fpls.2024.1322824

**Published:** 2024-01-24

**Authors:** Jiří Velechovský, Matěj Malík, Josef Baltazar Šenkyřík, Pavel Tlustoš

**Affiliations:** ^1^ Department of Agroenvironmental Chemistry and Plant Nutrition, Faculty of Agrobiology, Food and Natural Resources, Czech University of Life Sciences Prague, Prague, Czechia; ^2^ Department of Botany, Faculty of Science, Palacký University Olomouc, Olomouc, Czechia

**Keywords:** indoor-cultivation, cannabinoids, THC, *Cannabis sativa* L., soilless-cultivation, fertigation

## Abstract

Growing evidence underscores the role of nutrients and fertigation systems in soilless production, influencing medicinal cannabis biomass and secondary metabolite content. This study delves into the impact of enhanced nutrient regimes on the ‘ionome’ and its ramifications for biomass and cannabinoid production in medicinal cannabis, comparing two distinct fertigation systems: recirculation and drain-to-waste. Notably, we assess the optimal harvest time for maximizing profitability. In comparing the experimental variant with elevated levels of phosphorus (P), potassium (K), and iron (Fe) in the nutrient solution to the control variant, we observe distinct patterns in element composition across stems, leaves, and flowers, with significant differences between fertigation systems. Total nitrogen content was determined through the Kjeldahl method. Flame atomic absorption spectrometry (FAAS) and inductively coupled plasma optical emission spectrometry (ICP-OES) were employed for elemental analysis. Cannabinoid identification and quantification used high-performance liquid chromatography with a diode-array detector (HPLC/DAD). Followed statistical analyses included ANOVA and Tukey’s HSD test. Although the augmented nutrient regimen does not substantially increase plant biomass, interesting differences emerge between the two fertigation systems. The recirculation fertigation system proves more profitable during the recommended harvest period. Nonetheless, the altered nutrient regime does not yield statistically significant differences in final inflorescence harvest mass or cannabinoid concentrations in medicinal cannabis. The choice of fertigation system influences the quantity and quality of harvested inflorescence. To optimize the balance between the dry biomass yield of flowers and cannabinoid concentration, primarily total THC yield (sum of tetrahydrocannabinolic acid, Δ^9^-tetrahydrocannabinol, and Δ^8^-tetrahydrocannabinol), we propose the 11th week of cultivation as the suitable harvest time for the recirculation system. Importantly, the recirculation system consistently outperformed the drain-to-waste system, especially after the ninth week, resulting in significantly higher total THC yields. Enriched nutrition, when compared with control, increased THC yield up to 50.7%, with a remarkable 182% surge in the recirculation system when compared with the drain-to-waste system.

## 1 Introduction

The pharmaceutical industry faces unique challenges in large-scale cultivation and quality control of plant-based medications, especially with *Cannabis sativa* L., which is subject to varying international regulations (Chandra et al., 2017; Malík et al., 2021). Indoor cultivation has evolved through techniques like ‘sinsemilla’ (cultivating non-pollinated female plants), cuttings from superior mother plants, and hydroponic systems (Moeller and Lindholst, 2014). *In vitro* plant tissue culture techniques have emerged as a helpful approach (Hesami et al., 2021; Král et al., 2022; Šenkyřík et al., 2023), allowing for large-scale production of genetically identical plants in controlled laboratory conditions (Lata et al., 2016). These methods, combined with intensive breeding, have increased yields of female inflorescences and their THC (Δ^9^-tetrahydrocannabinol) levels (Toonen et al., 2006) and improved cannabinoid profile uniformity by altering plant architecture (Danziger and Bernstein, 2021). Indoor cultivation now incorporates automated systems (Malík et al., 2022), with hydroponics gaining popularity for its potential to yield high-quality plant material (Bouchard and Dion, 2009; Vanhove et al., 2011).

Environmental factors, particularly nutrient availability, significantly affect the quantity and quality of secondary metabolites such as cannabinoids and terpenes in cannabis (Janatová et al., 2018). Nutrients like nitrogen (N), phosphorus (P), potassium (K), and iron (Fe) play pivotal roles in plant growth and secondary metabolism (Bernstein et al., 2019b; Bevan et al., 2021; Kumar et al., 2021). Phosphorus, for instance, influences root growth, flower and seed production, and stem strength (Zheng, 2022) and affects terpene profiles in aromatic plants (Rioba et al., 2015). While inadequate P supplementation can lead to deficiency symptoms, excessive supply often accumulates in roots (Shiponi and Bernstein, 2021a; Llewellyn et al., 2023). The impact of increased phosphorus supplementation on medicinal cannabis varies across organs and compounds, with key cannabinoids remaining unaffected in upper flowers (Bernstein et al., 2019b; Shiponi and Bernstein, 2021b).

Potassium regulates water and nutrient transport, cell turgor pressure, disease resistance, stem strength, and inflorescence quality and yield (Oosterhuis et al., 2014; Johnson et al., 2022). It influences various secondary metabolites, such as phenolic compounds (Nguyen et al., 2010; Varo et al., 2022), flavonoids (Chrysargyris et al., 2017; Gaaliche et al., 2019), carotenoids (San Martín-Hernández et al., 2021), and organic acids (Naumann et al., 2020), but excessive potassium can reduce secondary metabolites like cannabinoids and terpenoids (Saloner and Bernstein, 2022). Optimizing K supply is crucial to maintaining medicinal cannabis desired functionality, yield, and secondary metabolite profiles. Iron facilitates oxidation-reduction and electron transfer reactions, activates enzymes, and is essential for photosynthesis and respiration (Briat et al., 2015; Zheng, 2022). Fe deficiency leads to chlorosis in young leaves, impaired root development, yield reduction, and compromised nutritional quality in crops (Kumar et al., 2021; Llewellyn et al., 2023). Fe concentrations in plant nutrition may have varying effects on secondary metabolite production (Shi et al., 2018; Chaouqi et al., 2023), but no specific study has explored the influence of Fe nutrition in indoor medicinal cannabis cultivation. Maintaining appropriate rootzone pH is crucial for nutrient availability, microorganism activity, and root development, affecting water and nutrient uptake (Zheng, 2021). The recommended pH range in hydroponic culture is typically between 5.5–6.0 (Velazquez, 2013), while soilless production suggests 5.5–6.5 (Zheng, 2022). If the pH in the soilless production drops below 5.5, there is a risk of toxicity due to excessive levels of manganese (Mn), while a pH higher than 6.5 can result in limited availability of essential elements such as P, Fe, and Mn for plant uptake (Balliu et al., 2021). pH levels affect nutrient availability and variations between recirculation and drain-to-waste systems, with recirculation systems exhibiting fluctuations (Malík et al., 2023).

This investigation examines the physiological and chemical reactions of medicinal cannabis plants when exposed to varying concentrations of phosphorus (P), potassium (K), and iron (Fe) in the nutrient solution. The study compares the results obtained under two distinct fertigation systems. In light of these objectives, the following hypotheses were put forth: (1) Alterations in the concentrations of P, K, and Fe in the nutrient solution can lead to different inflorescence yields of medicinal cannabis plants; (2) changes in the cannabinoid profile of indoor-cultivated cannabis plants; (3) these induced changes will exhibit a correlation with the content of macro-elements and micro-element (Fe) within different plant organs, including leaves, stems, and flowers; (4) furthermore, the induced changes will display variability between different nutrition systems, namely recirculation and drain-to-waste approaches.

## 2 Materials and methods

### 2.1 Parameters of the cultivation room

Cannabis plants were cultivated in a controlled environment within growing tables in a soilless system. A cultivation chamber has an area of 15 m^2^ (3 × 5 m), with the actual cultivation space delineated by four cultivation tables, covering a total area of 8 m^2^. The tables, measuring 2 m^2^ (1 × 2 m), were designated for individual experiments and featured independent 100-litre tanks for nutrient solutions. These tanks were constructed using food-grade inert plastic materials. Each table accommodated a maximum of 55 black conical, square pots made of polypropylene (PP). These pots had a volume of 3.45 liters and dimensions: top – 15 cm × 15 cm, base – 11.5 cm × 11.5 cm, height – 20 cm. Capillaries were used for fertigation, ensuring each plant received individualized watering through a needle applicator. The fertigation system was programmed to provide nine cycles per day, with each cycle lasting 60 seconds. During each cycle, 94 mL of the nutrient solution was delivered to each plant (equivalent to 846 mL per plant per day). In the recirculation system, the plant drainage was piped back to the storage tank. In the drain-to-waste system, the used nutrient solution was directed to a separate waste tank and not mixed with the original solution. Microclimate parameters, including relative humidity, temperature, and CO_2_ levels, were regulated and monitored by an air ventilation unit. A methane-burning generator facilitated CO_2_ enrichment in the growth environment (550 ppm). Six double-ended high-pressure sodium lamps provided illumination with a power output of 1,000 W, delivering a suitable light spectrum for plant growth. The lamps generated a photosynthetic photon flux density (PPFD) of 1,029 μmol·m^−2^·s^−1^, with a total power of 6,000 W. The light conditions were continuously recorded using a data logger, capturing measurements every minute.

### 2.2 Plant material and cultivation conditions

The plant material utilized in this study consisted of cuttings (apical parts with at least 3 fully expanded leaves) obtained from *C*. *sativa* L. ‘McLove’ mother plants (Atherton and Li, 2023). These plants belong to chemotype I, characterized by a high ratio of Δ^9^-tetrahydrocannabinolic acid:cannabidiolic acid (THCA : CBDA > 1.0) (Handbook of cannabis, 2016). The mother plants were carefully maintained in a dedicated separate growth chamber under controlled conditions. A total of 220 cuttings were prepared. These cuttings were then cultivated for a period of 21 days in rock-wool cubes measuring 4 × 4 cm. Once the cuttings had developed roots, they were transferred to a separate cultivation room and placed in 3.45-liter pots filled with three liters of expanded clay growing medium. During the vegetative phase, the light regime consisted of 18 hours of light and 6 hours of darkness, with a temperature of 25°C, a relative humidity of 60%, and a CO_2_ concentration of 550 ppm (1.065 mg·L^−1^). During the dark phase, the temperature was lowered to 22°C while maintaining the same humidity level. The vegetative phase lasted 7 days, after which the cultivation conditions were adjusted for the generative phase. During the generative phase, the light period was set to 12 hours of light and 12 hours of darkness. The temperature and CO_2_ concentration remained the same as during the vegetative phase, while the relative humidity was reduced to 40%. Starting from the 10th week, the plants were irrigated only with demineralized (DM) water.

### 2.3 Treatments

**Figure 1:**
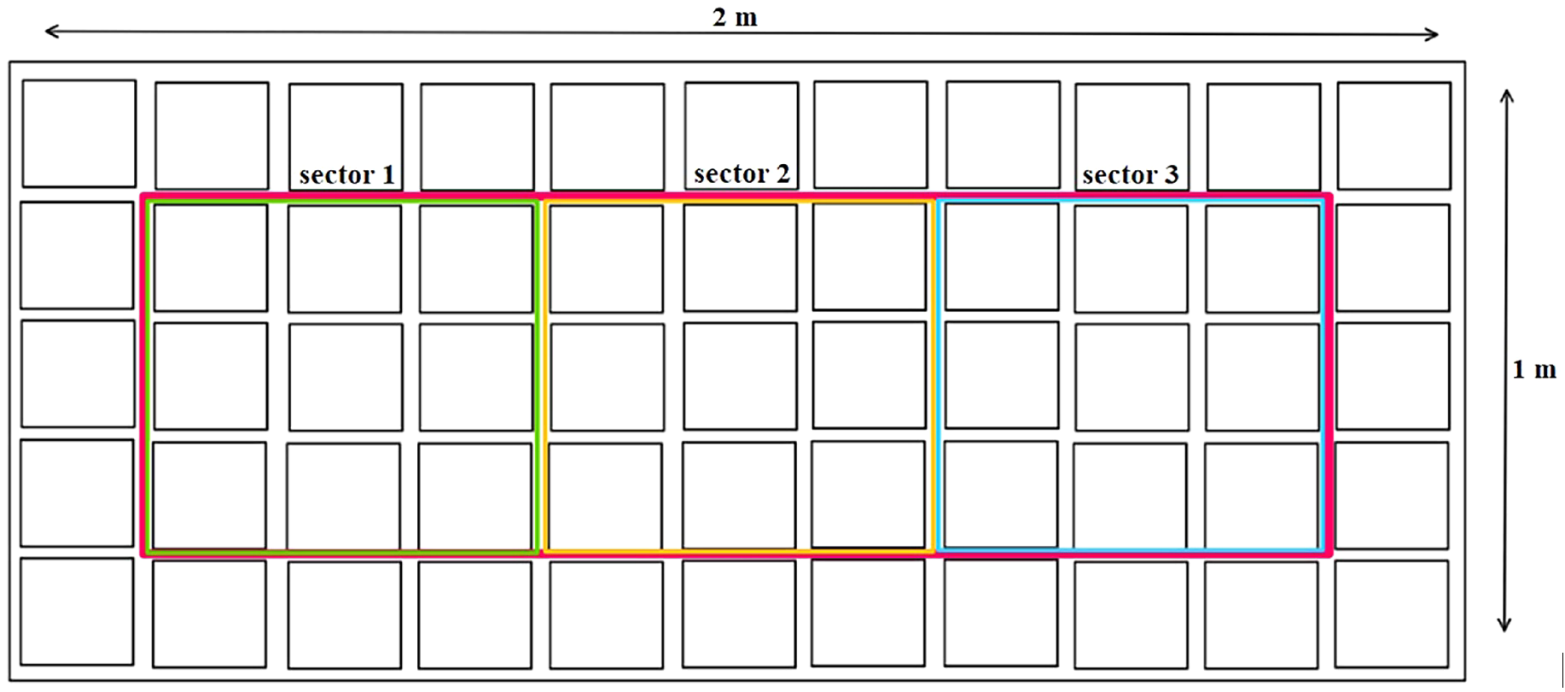
Plant sampling method.

In the cultivation room, the plants were divided into 4 tables, each containing 55 plants (27.5 plants/m^2^), as shown in **Figure 1**. Plants were grown in two fertigation systems. The first cycle (1C) involved the recirculation of the nutrient solution, while the second cycle (2C) employed the drain-to-waste system. At the same time, the plants were divided into two groups: control treatment (CN) and enhanced treatment (ET). Compared to CN, the ET underwent an augmented nutritional regime. For the 1C, the nutrient solution was prepared by mixing DM water every 7 days, starting from the experiment’s first day. Since the 10^th^ week, plants were irrigated solely with DM. Solution pH was adjusted to 5.9, and EC values were recorded immediately after preparing the fresh solution and remeasured on the last day before its replacement. A sample of the nutrient solution was collected after each preparation. For the 2C, everything remained the same as for 1C, except the stock solution was prepared after depleting the stock tank, approximately every 2nd to 3rd day. Nutrient content was increased according to the age of plants. **Table 1** presents the composition of the nutrient solution used for the CN as determined through measurement, and descriptions of the provided nutrients for ET can be found in **Table 2**. In contrast to the CN variant, the content of P, K, and Fe increased by an average of 83% P, 39% K, and 860% Fe from the 5th week to the 9th week in the ET variant.

**Table 1:** Composition of control treatment (CN) nutrient solution (mg·L^−1^).

Week	1	2	3	4	5	6–9	10–13
**N**	101 ± 1.6	116 ± 1.9	130 ± 1.8	150 ± 1.9	130 ± 1.8	150 ± 1.9	DM
**P**	32. ± 0.8	39 ± 0.8	44 ± 0.6	52 ± 0.8	44 ± 0.6	52 ± 0.8	DM
**K**	125 ± 1.9	151 ± 1.4	173 ± 1.9	193 ± 1.6	173 ± 1.9	193 ± 1.6	DM
**Ca**	99 ± 1.3	119 ± 1.4	132 ± 1.4	146 ± 1.2	132 ± 1.4	146 ± 1.3	DM
**Mg**	25 ± 0.4	31 ± 0.4	35 ± 0.5	39 ± 0.5	35 ± 0.5	39 ± 0.5	DM
**S**	22 ± 0.3	27 ± 0.3	31 ± 0.3	35 ± 0.4	31 ± 0.3	35 ± 0.4	DM
**Fe**	0.9 ± 0.09	1.1 ± 0.09	1.2 ± 0.11	1.4 ± 0.08	1.2 ± 0.11	1.4 ± 0.08	DM
**Mn**	0.7 ± 0.07	0.7 ± 0.05	0.8 ± 0.08	0.9 ± 0.07	0.8 ± 0.08	0.9 ± 0.07	DM
**Zn**	0.2 ± 0.03	0.3 ± 0.03	0.3 ± 0.04	0.3 ± 0.03	0.3 ± 0.04	0.3 ± 0.03	DM
**Cu**	0.1 ± 0.01	0.1 ± 0.01	0.1 ± 0.01	0.1 ± 0.02	0.1 ± 0.01	0.1 ± 0.02	DM
**B**	0.1 ± 0.02	0.2 ± 0.01	0.2 ± 0.02	0.2 ± 0.02	0.2 ± 0.02	0.2 ± 0.02	DM
**Mo**	0.01 ± 0.00	0.02 ± 0.00	0.02 ± 0.00	0.02 ± 0.00	0.02 ± 0.00	0.02 ± 0.00	DM
**EC**	0.97 ± 0.01	1.19 ± 0.01	1.46 ± 0.01	1.74 ± 0.01	1.46 ± 0.01	1.74 ± 0.01	DM

DM, demineralized water.

**Table 2:** Composition of enhanced treatment (ET) nutrient solution with the elevated P, K, and Fe (mg·L^−1^).

Week	1	2	3	4	5	6–9	10–13
**N**	100 ± 0.9	115 ± 1.0	129 ± 0.1	150 ± 1.8	129 ± 1.7	150 ± 1.8	DM
**P**	32 ± 0.5	39 ± 0.4	42 ± 0.5	51 ± 0.9	92 ± 1.9	93 ± 1.9	DM
**K**	125 ± 1.6	143 ± 1.2	172 ± 1.9	194 ± 1.4	258 ± 2.5	266 ± 2.7	DM
**Ca**	98 ± 0.9	118 ± 1.2	132 ± 1.	146 ± 1.2	133 ± 1.1	144 ± 1.4	DM
**Mg**	25 ± 0.2	29 ± 0.4	35 ± 0.4	39 ± 0.4	33 ± 0.4	39 ± 0.7	DM
**S**	22 ± 0.1	26 ± 0.9	31 ± 0.4	34 ± 0.3	31 ± 0.4	35 ± 0.3	DM
**Fe**	0.9 ± 0.01	1.1 ± 0.06	1.3 ± 0.05	1.3 ± 0.08	12.3 ± 0.4	13.8 ± 0.9	DM
**Mn**	0.7 ± 0.06	0.7 ± 0.04	0.7 ± 0.09	0.9 ± 0.04	0.8 ± 0.06	0.9 ± 0.07	DM
**Zn**	0.2 ± 0.04	0.3 ± 0.03	0.3 ± 0.04	0.3 ± 0.02	0.3 ± 0.04	0.3 ± 0.03	DM
**Cu**	0.1 ± 0.01	0.1 ± 0.02	0.1 ± 0.01	0.1 ± 0.02	0.1 ± 0.01	0.1 ± 0.01	DM
**B**	0.1 ± 0.01	0.2 ± 0.01	0.2 ± 0.02	0.2 ± 0.01	0.2 ± 0.01	0.2 ± 0.02	DM
**Mo**	0.01 ± 0.00	0.02 ± 0.01	0.02 ± 0.00	0.02 ± 0.00	0.02 ± 0.01	0.02 ± 0.01	DM
**EC**	0.96 ± 0.01	1.20 ± 0.01	1.45 ± 0.02	1.54 ± 0.01	2.05 ± 0.02	2.34 ± 0.06	DM

DM, demineralized water.

### 2.4 Plant material sampling

For each treatment and cycle, three plants were harvested every 7 days throughout the entire vegetation period. One plant was randomly selected from each highlighted sector (1–3), as shown in **Figure 1**. Additionally, a random plant from the edge (outside the sectors) was transferred to an empty space within each sector. The sampled plants were weighed fresh as a whole and then divided into leaves, stems, and flowers, which were weighed separately. Subsequently, the materials were dried at a constant moisture level of 8–10% at 25°C and reweighed. To determine the dry matter, a reference amount of each plant part was dried at 105°C until a constant weight was achieved. Just before analysis, the plant parts were homogenized. The flowers (including leaves until the 4th week) were frozen using liquid nitrogen and then ground using a mortar and pestle. The dried leaves (from the 5th week) and stems were ground using a grinder.

### 2.5 Dry decomposition and elemental analysis

The leaves, stems, and flowers were individually analyzed to determine the content of macroelements (excluding nitrogen), microelements, and trace elements in the plant samples. The plant biomass, which had been weighed and homogenized, was placed in a beaker and covered with a watch glass. The beaker was then placed on a hotplate set at 160°C, and the temperature was gradually increased to 350°C over a period of 4 hours to facilitate decomposition of the samples. Subsequently, the samples were transferred to a muffle furnace and maintained at a temperature of 450–500°C for 12 hours, as described in previous studies (Miholová et al., 1993). After cooling, 1 mL of 65% HNO_3_ was added to the beakers, which were then placed on a hot plate set at 120°C for 60 minutes. Following this step, the samples were annealed for 90 minutes in an oven at 500°C and suspended in 1.5% HNO_3_ with stirring using an ultrasonic bath. Elemental analysis of the samples was conducted using flame atomic absorption spectrometry (FAAS) on a Varian 280FS instrument (Varian, Australia), coupled with inductively coupled plasma optical emission spectrometry (ICP-OES) performed on a Varian Vista-PRO (Varian, Australia), (Mester and Sturgeon, 2003).

### 2.6 Determination of nitrogen content in plant material

The Kjeldahl method was employed to determine the total nitrogen content in the plant material, encompassing both organic and ammonia nitrogen. Approximately 0.50 g of the sample was weighed for analysis, followed by mineralization. The mineralization process was conducted in glass vials, where 2 g of catalyst (a mixture of 100 g K_2_SO_4_, 1 g CuSO_4_, 0.1 g Se) and 10 mL of concentrated sulfuric acid (H_2_SO_4_) were added to the sample. Decomposition took place for 90 minutes at a temperature of 420°C. After mineralization, the samples were prepared for distillation. Within the apparatus, 20 mL of distilled water was automatically added to the vial, followed by distillation into H_3_BO_3_, allowing for the determination of the total nitrogen content in the sample. The content was ascertained through titration using HCl (0.5 mol·L^−1^) and subsequently measured using the Gerhardt Vapodest 30s instrument (Königswinter, Germany) (Baker and Thompson, 1992).

### 2.7 Identification and quantification of cannabinoids

An optimized method of dynamic maceration was employed to identify and quantify cannabinoid content using HPLC/DAD (high-performance liquid chromatography with a diode-array detector), as described by Patel et al. (2017). Subsequently, 0.150 g of the sample was weighed into a 50 mL beaker, and 5 mL of solvent (96% ethanol) was added. The sample was then dynamically macerated for 60 minutes, filtered using a Morton filtration device (P16 porosity), and the filtrate was transferred to 50 mL vials. The same solvent in the same volume was added again to the initial 0.150 g of plant material, and the sample was dynamically macerated for another 60 minutes. This process was repeated three times, resulting in a composite sample derived from the three-phase dynamic maceration process in a 1:100 (V:W) ratio. This sample was diluted 20 times (using 96% ethanol), filtered through a syringe filter (0.22 μm), transferred to vials, and prepared for analysis in this form. Samples of the extracts were introduced into a high-performance liquid chromatography system equipped with diode array detection (HPLC-DAD; Agilent 1,260, Agilent Technologies Inc., United States), utilizing a Luna^®^ 1C8 column (2) with dimensions of 250 × 3 mm and a particle size of 3 μm (Phenomenex, United States). The mobile phase employed was an isocratic mixture of acetonitrile/H_2_O (31:9, v/v) containing 0.1% HCOOH (v/v) and 0.1 mol/L NH_4_COOH (without pH adjustment). The flow rate was set at 0.55 mL/min, the temperature at 37°C, the sample injection volume at 8 μl, and UV detection was performed at 275 nm (Križman, 2020). To ensure accuracy, the instrument was externally calibrated using Δ^9^-tetrahydrocannabinol (Δ^9^-THC), Δ^8^-tetrahydrocannabinol (Δ^8^-THC), cannabigerolic acid (CBGA), cannabigerol (CBG) and cannabinolic acid (CBNA) ranging from 0.3 to 10 mg·L^−1^, and THCA ranging from 0.3 to 100 mg·L^−1^ (Sigma-Aldrich, Czech Republic) as reference standards. Data analysis was carried out using OpenLAB CDS software, ChemStation Edition, Rev. C.01.5.

### 2.8 Statistical analyses

The data were subjected to analysis of variance (one-way ANOVA) followed by Tukey’s Honestly Significant Difference (HSD) test using IBM SPSS Statistics software (version 25, 2017, Chicago, Illinois, United States).

## 3 Results

**Figure 2:**
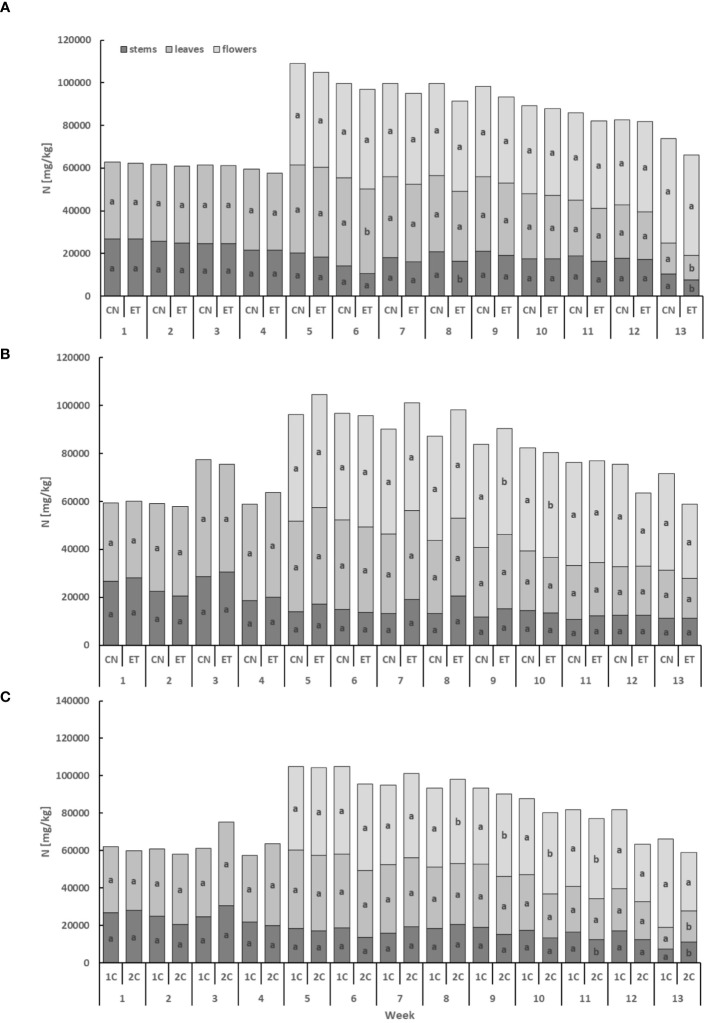
Concentration of nitrogen in the dry weight among the organs of indoor-cultivated cannabis plants as affected by augmented nutritional regime in recirculation and drain-to-waste nutrient cycles. N concentration of control (CN) and enhanced treatment (ET) under the augmented nutritional regime in recirculation (1C) growing cycle **(A)**, in drain-to-waste (2C) growing cycle **(B)**, ETs in 1C and 2C **(C)** in stems, leaves, and flowers. Data are means (n = 3). The different small letters inside the bars represent significant differences within the individual plant organs (stems, leaves, and flowers) between the variants in a particular week, according to Tukey’s HSD test at α = 0.05.

Implementing an enhanced nutritional regime and using different fertigation systems (1C and 2C) resulted in alterations in the plant nutrient composition of cannabis plants. Simultaneously, the utilization of two distinct systems resulted in variations in the pH levels of the nutrient solution. In the 2C system, a consistent pH value of 5.9 was upheld throughout the cycle, whereas in the 1C system, pH exhibited fluctuations within the range of 5.9–6.95. The nitrogenous compounds exhibited the lowest concentration in the stems, while the flowers displayed the highest concentration (**Figure 2**). Comparing the leaves and flowers of CN and ET plants under the augmented nutritional regime in the 1C cycle, statistically significant differences in N concentrations were observed only twice in the leaves (6th and 13th week) and twice in the stems (8th and 13th week). Notably, the most significant variation in N concentrations between CN and ET within the 1C system occurred in the stems during the 13th week, with a difference of 28% (CN: 10.44 mg·g^−1^, ET: 7.5 mg·g^−1^; **Figure 2A**). In contrast, N concentrations in the stems and leaves of CN and ET plants in 2C did not significantly differ in any of the tested weeks. Nevertheless, the concentration of N in flowers was significantly different in weeks 9 and 10 in the 2C regime. N concentrations exhibited over all the highest differences between 1C and 2C of ETs under the augmented nutritional regime, primarily noticeable in the flowers from week 8 to 11. During the last week, significant differences were observed only in the stems and leaves. The most significant variation in N concentrations between fertigation systems in ETs was observed during the 13th week, with a difference of 42% (21% between CNs) in the leaves (1C: 42.18 mg·g^−1^, 2C: 45.28 mg·g^−1^; **Figure 2C**).

**Figure 3:**
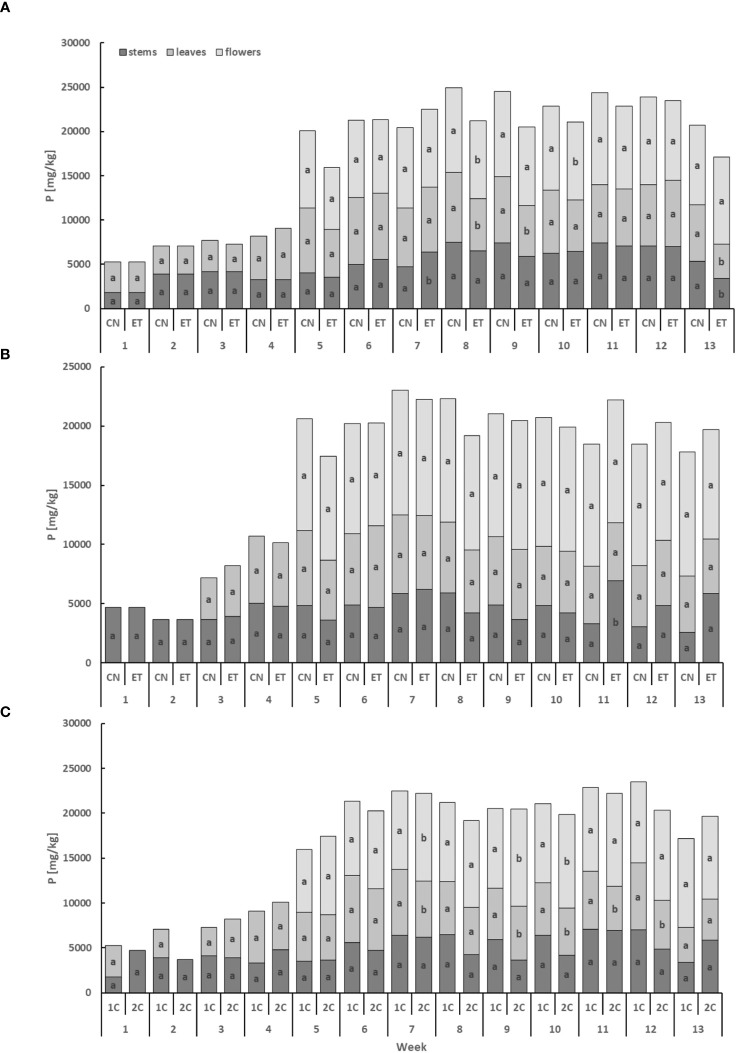
Phosphorus concentration in the dry weight among the organs of indoor-cultivated cannabis plants as affected by the augmented nutritional regime in recirculation and drain-to-waste nutrient cycles. P concentration of control (CN) and enhanced treatment (ET) under the augmented nutritional regime in recirculation (1C) growing cycle **(A)**, in drain-to-waste (2C) growing cycle **(B)**, ETs in 1C and 2C **(C)** in stems, leaves, and flowers. Data are means (n = 3). The small letters inside the bars represent significant differences within the plant organs (stems, leaves, and flowers) between the variants in a particular week, according to Tukey’s HSD test at α = 0.05.

The phosphorus (P) content exhibited lower levels in the leaves and stems compared to the flowers, displaying a cumulative pattern over time (**Figure 3**). In the stems, the P concentration of CN and ET in the 1C cycle significantly differed only in weeks 7 and 13. Significant differences were observed in the leaves in weeks 8, 9, and 13, while in the flowers, differences were found in weeks 8 and 10. Notably, the most significant variation in P concentration between nutritional treatments was observed in the leaves during the 13th week, with a difference of 63% (CN: 63.48 mg·g^−1^, ET: 38.9 mg·g^−1^; **Figure 3A**). In contrast to the 1C cycle, the P concentration of CN and ET in 2C plants showed significant differences only in the 11th week in the stems, with a difference of 108% (CN: 33.3 mg·g^−1^, ET: 69.15 mg·g^−1^; **Figure 3B**). Furthermore, the P concentration in the leaves and flowers of ET plants also varied between the 1C and 2C cycles. Significant differences were observed in the leaves in weeks 7, 9, 10, 11, and 12, while in the flowers, differences were found in weeks 7, 9, and 10. However, when comparing these two regimes, no statistically significant differences were observed in the P concentration of stems. The most notable differences in P concentrations between the 1C and 2C cycles in ETs were observed during the 11th week, with a difference of 31.6% (36% between CNs) in the leaves (1C: 64.87 mg·g^−1^, 2C: 49.31 mg·g^−1^; **Figure 3C**).

**Figure 4:**
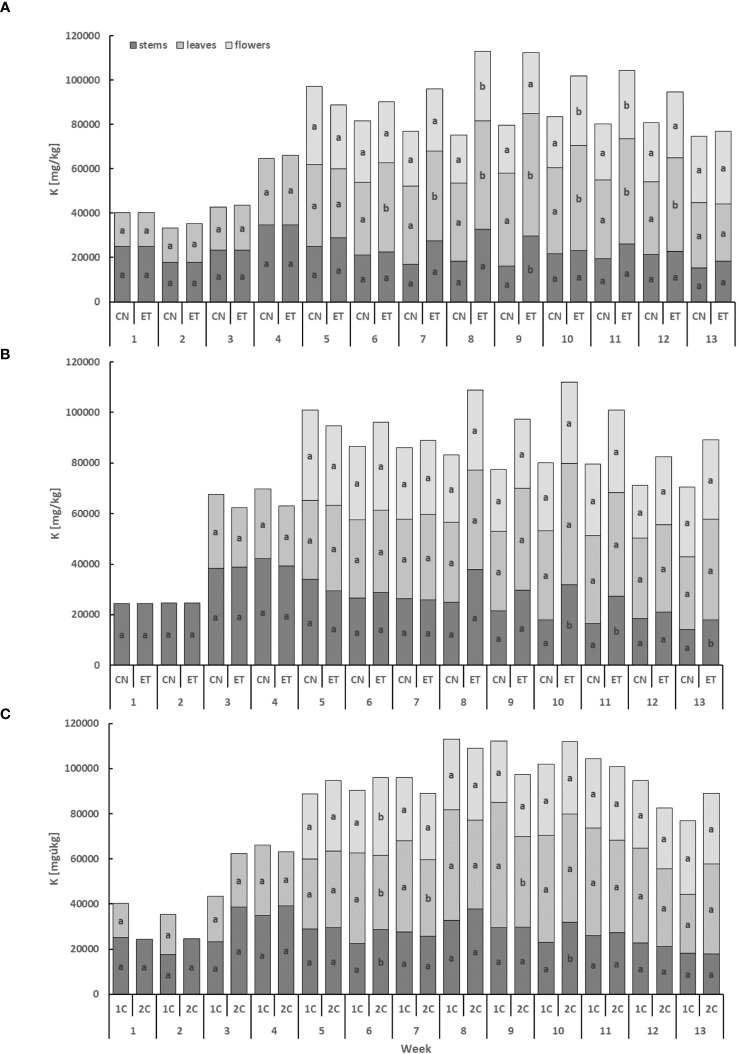
Potassium concentration in the dry weight among the organs of indoor-cultivated cannabis plants as affected by the augmented nutritional regime in recirculation and drain-to-waste nutrient cycles. K concentration of control (CN) and enhanced treatment (ET) under the augmented nutritional regime in recirculation (1C) growing cycle **(A)**, in drain-to-waste (2C) growing cycle **(B)**, ETs in 1C and 2C **(C)** in stems, leaves, and flowers. Data are means (n = 3). The small letters inside the bars represent significant differences within the plant organs (stems, leaves, and flowers) between the variants in a particular week, according to Tukey’s HSD test at α = 0.05.

The potassium (K) content displayed the lowest levels in the stems and the highest levels in the leaves (**Figure 4**). In the leaves, the concentration of K for CN and ET with the 1C cycle started to exhibit significant differences from the 6th week until the 12th week. Significant differences were observed in the flowers in weeks 8, 10, and 11, while in the stems, differences were found only in week 9. Notably, the most significant variation in K concentration between CN and ET was observed in the stems during the 9th week, with a difference of 45.5% (CN: 16.12 mg·g^−1^, ET: 29.57 mg·g^−1^; **Figure 4A**). In contrast to the 1C cycle, the concentration of K in the 2C cycle of CN and ET plants showed significant differences only in the stems in weeks 10, 11, and 13. The most substantial differences in K concentrations between nutritional treatments were observed in the stems during the 10th week, with a difference of 77% (CN: 18.02 mg·g^−1^, ET: 31.88 mg·g^−1^; **Figure 4B**). Likewise, the K concentration also varied between the 1C and 2C cycles of ETs, with significant differences occurring in weeks 6, 7, 9, and 10. The most notable differences in K concentrations between 1C and 2C in ETs were observed in the stems during the 10th week, with a difference of 38.8% (20% between CNs) (1C: 23.03 mg·g^−1^, 2C: 31.88 mg·g^−1^; **Figure 4C**).

**Figure 5:**
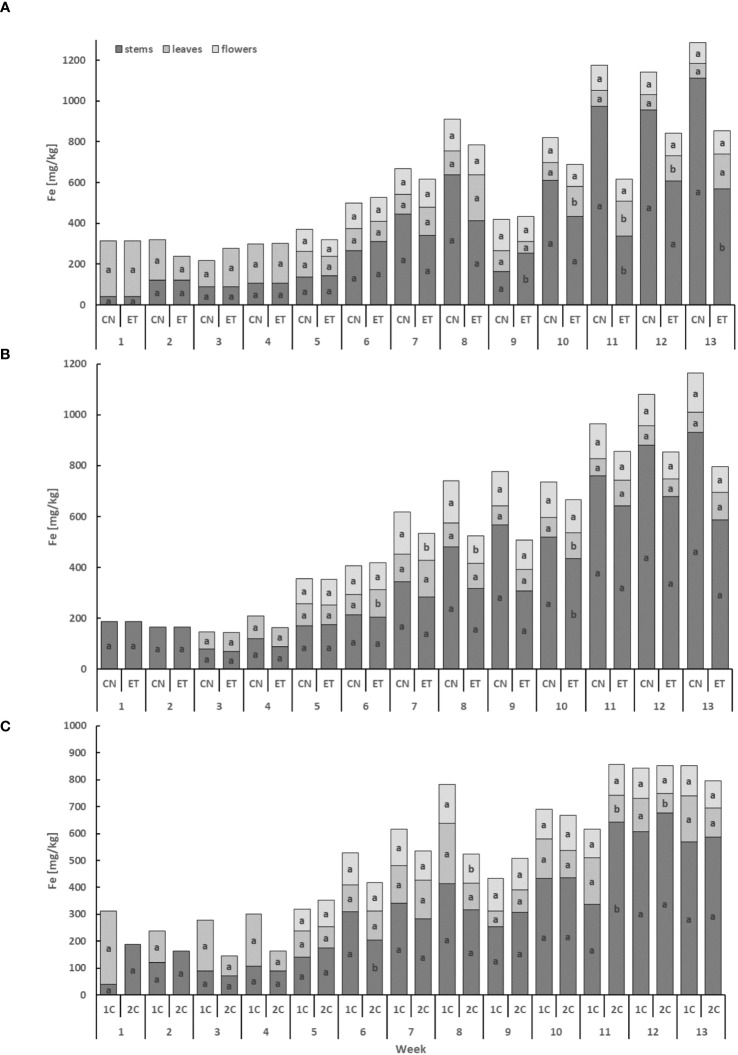
Iron concentrations in the dry weight among the organs of indoor-cultivated cannabis plants as affected by the augmented nutritional regime in recirculation and drain-to-waste nutrient cycles. Fe concentration of control (CN) and enhanced treatment (ET) under the augmented nutritional regime in recirculation (1C) growing cycle **(A)**, in drain-to-waste (2C) growing cycle **(B)**, ETs in 1C and 2C **(C)** in stems, leaves, and flowers. Data are means (n = 3). The small letters inside the bars represent significant differences within the plant organs (stems, leaves, and flowers) between the variants in a particular week, according to Tukey’s HSD test at α = 0.05.

The iron (Fe) content exhibited the lowest levels in leaves and the highest levels in stems, displaying a cumulative trend over time (**Figure 5**). In the stems, the concentration of Fe for CN and ET with the 1C cycle showed significant differences in weeks 9, 11, and 13. Notably, the most significant variation in Fe concentration between CN and ET was observed in the stems during the 11th week, with a difference of 188.7% (CN: 337.2 mg·kg^−1^, ET: 955.8 mg·kg^−1^; **Figure 5A**). In contrast to the 1C cycle, the concentration of Fe in the stems of CN and ET plants in the 2C cycle exhibited significant differences only in week 10, in the leaves merely in weeks 6 and 10, and in the flowers from the 7th until the 8th week. The most substantial difference in Fe concentrations between CN and ET of 2C plants was observed in the flowers during the 8th week, with a difference of 55% (CN: 167.3 mg·kg^−1^, ET: 108.2 mg·kg^−1^; **Figure 5B**). Besides, the Fe concentration varied between the 1C and 2C cycles of ETs, with significant differences observed in the stems during weeks 6 and 11, in the leaves during weeks 11 and 12, and in the flowers during week 8. The most notable difference in Fe concentrations between 1C and 2C in ETs was observed in the stems during the 11th week, with a difference of 90.6% (11% between CNs) (1C: 337.2 mg·kg^−1^, 2C: 642.6 mg·kg^−1^; **Figure 5C**).

**Figure 6:**
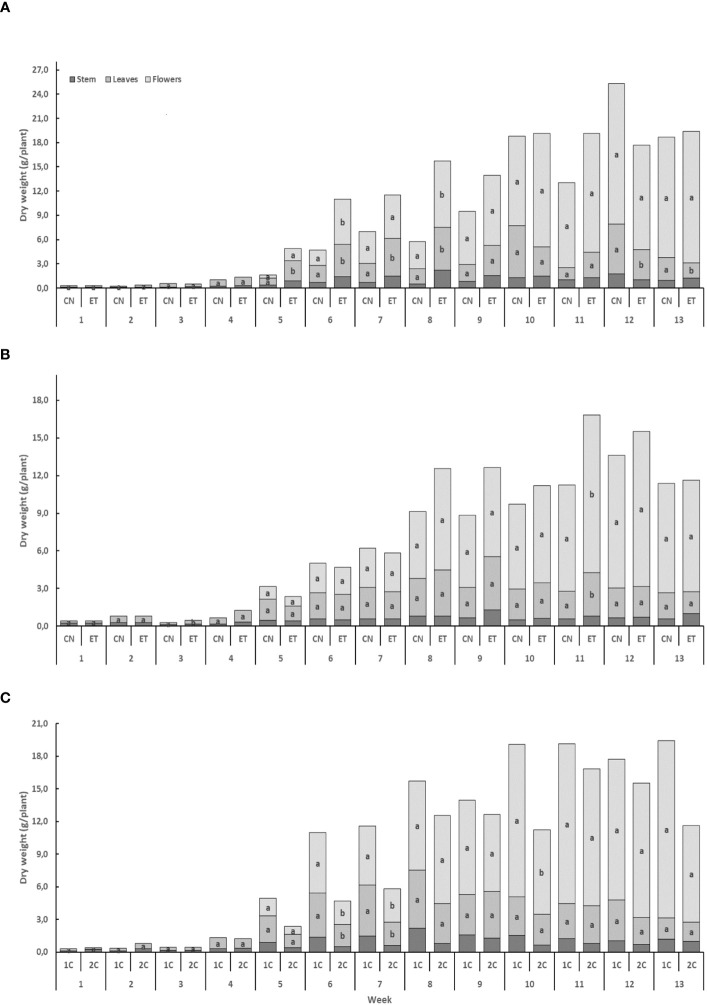
The effect of the augmented nutritional regime and growing nutritional cycle on indoor-cultivated cannabis plant biomass. Dry biomasses of stems, leaves, and flowers in control (CN) and enhanced treatment (ET) plants with augmented nutritional regime in recirculation (1C) growing cycle **(A)**, in drain-to-waste (2C) growing cycle **(B)**, ETs in 1C and 2C **(C)**. Data are means (n = 3). The different small letters inside the bars and small bold letters above the bars represent significant differences within the indoor-cultivated cannabis plant organs (leaves and flowers) and the whole plant biomass between the variants in a particular week according to Tukey’s HSD test at α = 0.05.

Implementing an augmented nutritional regime in two distinct nutritional cycles led to noticeable effects on the growth of cannabis plants. The biomass increase was relatively slow during the initial four weeks, but a significant acceleration was observed from week 5 onwards. Notably, the highest weekly dry weight gain was observed in the flowers (**Figure 6**). In the 1C cycle, the biomass increases in stems, leaves, and flowers for both CN and ET plants was nearly identical until week 4. However, from week 5 to 8, some differences in leaf and flower biomass emerged (**Figure 6A**), and in the 13th week, a statistically significant disparity in flower dry weight was noted. In contrast, the dry weight of stems, leaves, and flowers in the 2C cycle did not exhibit significant increases for CN and ET plants, except for the dry weight of leaves in the 3rd week and the dry weight of flowers in the 11th week. ET plants reached their maximum dry biomass at week 11, while CN plants achieved it by week 12 (**Figure 6B**). Biomass variations were also observed between the 1C and 2C cycles in ET plants, commencing from week 6 (**Figure 6C**).

**Figure 7:**
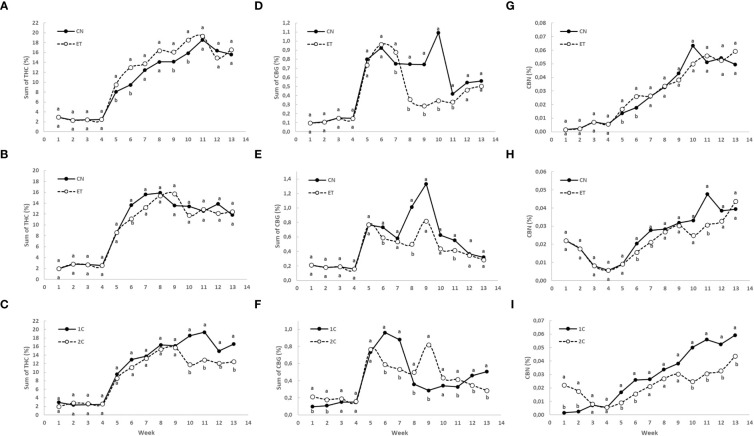
Concentrations of total THC (sum of tetrahydrocannabinolic acid, Δ^9^-tetrahydrocannabinol, and Δ^8^-tetrahydrocannabinol), total CBG (sum of cannabigerolic acid and cannabigerol), and cannabinolic acid (CBNA) in the flowers of indoor-cultivated cannabis plants as affected by augmented nutritional regime and nutrient cycle. The sum of THC concentration in control (CN) and enhanced treatment (ET) plants with augmented nutritional regime in recirculation (1C) growing cycle **(A)**, in drain-to-waste (2C) growing cycle **(B)**, ETs in 1C and 2C **(C)**. The sum of CBG concentration of control (CN) and enhanced treatment (ET) with augmented nutritional regime in recirculation (1C) growing cycle **(D)**, in drain-to-waste (2C) growing cycle **(E)**, ETs in 1C and 2C **(F)**. CBNA concentration of control (CN) and enhanced treatment (ET) with augmented nutritional regime in recirculation (1C) growing cycle **(G)**, in drain-to-waste (2C) growing cycle **(H)**, ETs in 1C and 2C **(I)**. The whole inflorescence of the plant was analyzed. Data are means (n = 3). Different bold small letters represent significant differences in cannabinoid concentration between the variants in a particular week according to Tukey’s HSD test at α = 0.05.

The implementation of an augmented nutritional regime had statistically significant impact on the concentration of total THC (sum of Δ^9^-tetrahydrocannabinol, Δ^8^-tetrahydrocannabinol, and tetrahydrocannabinolic acid) total CBG (sum of cannabigerolic acid and cannabigerol), and CBNA in the flowers of medicinal cannabis plants, with distinct patterns observed for different fertigation systems and nutrition treatments (**Figure 7**). The total THC and CBNA concentration curves exhibited similar trends within the same growing cycle and treatment. At the same time, total THC yield (g/m^2^) was evaluated, where there was a clear trend in favor of ET across all comparisons.

**Figure 8:**
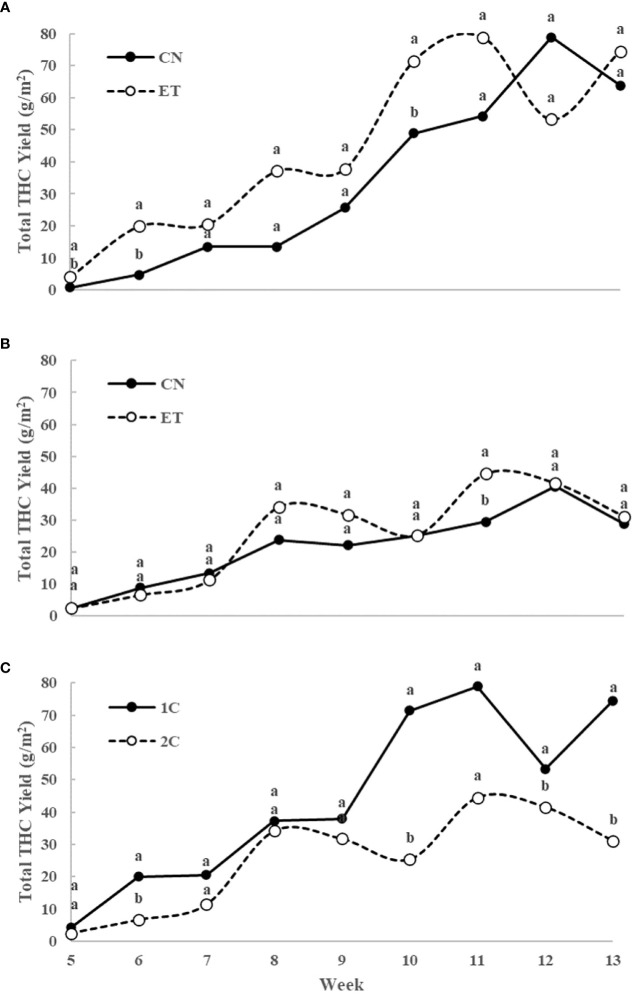
Total yield of THC (sum of tetrahydrocannabinolic acid, Δ^9^-tetrahydrocannabinol, and Δ^8^-tetrahydrocannabinol) per one square meter of indoor-cultivated cannabis plants as affected by augmented nutritional regime and nutrient cycle. Total THC yield in control (CN) and enhanced treatment (ET) plants with augmented nutritional regime in recirculation (1C) growing cycle **(A)**, in drain-to-waste (2C) growing cycle **(B)**, ETs in 1C and 2C **(C)**. Data are means (n = 3). Different bold small letters represent significant differences in cannabinoid concentration between the variants in a particular week according to Tukey’s HSD test at α = 0.05.

Initially, the concentration of total THC in leaves and flowers almost stagnated in both treatments until week 4. However, from week 5 onwards, substantial growth with significant differences in total THC concentration was observed because only flowers were analyzed (**Figures 7A-C**). In the 1C cycle, significant differences in total THC concentration between CN and ET treatments occurred in weeks 5, 6, 9, and 10. The maximum concentrations were reached at week 11 for both CN (18.5%) and ET (19.4%) (**Figure 7A**). In contrast, in the 2C cycle, significant differences in total THC concentration between CN and ET treatments were observed only in the 6th week. CN reached its peak concentration at week 8 (15.9%), while ET reached its peak at week 9 (15.7%) (**Figure 7B**). Significant differences in total THC levels between 1C and 2C of ET treatments were observed from week 10 until the end of the experiment, where the 1C overperformed 2C (**Figure 7C**). The yield of this dominant cannabinoid (THC) showed significant differences in the 1C regime in favor of the ET treatment. Specifically, there was an increase in yield per square meter in weeks 5, 6 and 10 by 402%, 315% and 46%, respectively (**Figure 8A**). In drain to waste system (2C), this trend was similar. Still, a statistically significant difference was found only in the eleventh week, when there was an increase in THC yield in the ET variant by 50.7% (**Figure 8B**). An interesting finding is that when comparing the 1C and 2C regimes with the ET variant, there was an overall lower THC yield with the drain-to-waste system (2C). Specifically, there was a statistically significant difference in weeks 6, 10, 12 and 13 when there was a decrease in the THC yield of the 2C system to 6.6%, 25.3%, 41.5% and 31.1% of the THC yield value of the 1C recirculation system (**Figure 8C**).

For total CBG, the concentration in leaves and flowers nearly remained stagnant until week 4, with a notably lower concentration compared to THC by more than an order of magnitude. However, a significant increase in total CBG concentration was observed in week 5, focusing only on the flowers (**Figures 7D-F**). In the 1C cycle, the concentrations of total CBG in CN and ET treatments began to differ significantly from week 7 until week 11 and peaked at week 10 for CN (1.1%) and week 6 for ET (0.9%). The most significant differentiation between CN and ET occurred at week 10, with a difference of 218% in favor of CN. Following the peak concentrations, a foretellable decrease in concentration was observed, followed by a slight increase in the last three weeks. In the 2C cycle, significant differences in total CBG concentration between CN and ET treatments were observed only in weeks 6, 8, and 11. The maximum concentrations were reached at week 9 for both CN (1.3%) and ET (0.8%) (**Figure 7E**). Significant differences in total CBG levels between 1C and 2C of ET treatments were observed in weeks 1, 2, and from week 6 except the 10th week until the end of the experiment (**Figure 7F**).

Regarding CBNA concentration, significant variations between CN and ET treatments in the 1C cycle were observed in weeks 5 and 6. CBNA concentration peaked at week 10 for CN (0.06%) and week 13 for ET (0.06%) (**Figure 7G**). In the 2C cycle, significant differences in CBNA concentration between CN and ET treatments were observed in weeks 6, 7, and 11. CN reached its maximum concentration (0.04%) in 2C at week 11, with a significant difference of 55%, while ET reached its maximum in the 13th week (**Figure 7H**). CBNA concentrations between 1C and 2C of ET treatments showed significant differences in the first two weeks, followed by nearly identical concentration levels in weeks 3 and 4. From week 5 onwards, a steady growth trend was observed, with 1C outperforming 2C. Significant differences were observed in weeks 1, 2, 5, and 6 and from week 10 until the end of the experiment. As mentioned, the CBNA concentrations in both ET variants reached their maximum at week 13, with a significant difference of 34% (**Figure 7I**).

## 4 Discussion

The present study focused on investigating the effects of an enhanced nutritional regime and different fertigation systems on the ionome composition, biomass, and cannabinoid content of indoor-cultivated cannabis plants. The results indicate substantial variations in N, P, K, and Fe concentrations in different plant parts under the augmented nutritional regime. These alterations could be attributed to nutrient uptake, translocation, and allocation modifications resulting from the introduced nutritional variations. Undoubtedly, nutrition plays a pivotal role in shaping the development, functionality, and metabolic processes of various plant organs and tissues (Shiponi and Bernstein, 2021b; De Prato et al., 2022). Existing knowledge has extensively documented the ideal thresholds of specific macronutrients, including N, P and K, along with micronutrients like Fe, necessary for ensuring the normal growth and functioning of both root systems and above-ground biomass (Shiponi and Bernstein, 2021a; Shiponi and Bernstein, 2021b; Malík et al., 2022; Saloner and Bernstein, 2022; Llewellyn et al., 2023), as well as the production of valuable secondary metabolites in medicinal cannabis plants (Caplan et al., 2017; Bernstein et al., 2019b; Shiponi and Bernstein, 2021b; Saloner and Bernstein, 2022). It is crucial to emphasize that nutrient solution fertigation was provided to the plants only up to the ninth week of cultivation. Subsequently, from the ninth week onwards, the plants received fertigation solely with deionized (DM) water. Consequently, the plants relied solely on their accumulated nutrient reserves during this period. Modifications in the nutrient solution’s P, K, and Fe concentrations within two distinct nutrient cycles undeniably exerted discernible impacts on the levels of macro and micro-elements in cannabis plants’ above-ground organs. While the N concentration remained unchanged between the CN and ET systems, some statistically significant differences in N distribution among the above-ground organs of cannabis plants were observed within each fertigation system. The composition and proportional disbalance of nutrients in nutrient solutions have been recognized as factors capable of influencing the profiles of cannabinoids and terpenes in cannabis. These observed variations are likely linked to the complex interplay between nutrient levels in the solution and the growth of the plant. The different fertigation systems employed in this study also exhibited discernible effects on the concentrations of both macro- and microelements within the tissues of cannabis plants. It should be emphasized that the interactions between cations and anions of nutrients during root cell membrane transport have been extensively documented in the scientific literature (Meychik and Yermakov, 2001; Roberts, 2006; Langenfeld et al., 2022).

Nevertheless, variations in the pH of the nutrient solution, such as those observed in the RS (1C), and the increased nutrient quantity provided in the DS (2C), have the potential to influence the availability of specific nutrients and, consequently, the physiological and metabolic reactions within the plants (Malík et al., 2023). Furthermore, a more pronounced variation in N concentrations was evident when comparing the two distinct fertigation systems, which aligns with our study. Nitrogen (N) plays a vital role in organs with high metabolic activity, such as leaves and flowers, where it is a crucial component of proteins and compounds related to energy metabolism. As a result, N tends to accumulate in smaller quantities in structural compartments with lower metabolic activity, such as stems (Marschner and Marschner, 2012). In our investigation comparing the 2C and 1C systems, the 2C system consistently exhibited higher N concentrations in flowers during weeks 8 to 11 (an average 8.7% increase in favor of the 2C system, with values ranging from 6.3% to 11.6%). This contrasts with the findings of Malík et al. (2023), where they observed higher levels of N in plant tissues in the recirculation systems. In our case, we presume that the more frequent nutrient replenishment (2–3 times a week) in the drain-to-waste system resulted in more significant nutrient accumulation in both the above-ground biomass and the roots. Subsequently, when nutrition was stopped, and only DM water was irrigated, there was translocation from the roots to the above-ground biomass. Also, another hypothesis could be that some of the nutrients that were supplied in addition to the ET variant (P, K, or Fe) could be limiting in the recirculation system. Therefore, when it was supplied more in drain-to-waste, other nutrients (in this case, N) could subsequently be more incorporated into tissues (Ågren et al., 2012; Asao, 2012).

As expected, alterations in the concentrations of P, K, and Fe in the nutrient solution in some observations significantly impacted the concentrations of these elements in the above-ground organs of medicinal cannabis plants. Notably, in the 1C regime, differences between CN and ET were more pronounced than in the 2C regimen. This observation can be attributed to the fluctuation and pH increase in the 1C compared with the stable pH in the 2C regime (Malík et al., 2023). Surprisingly, despite doubling the concentration of P in the nutrient solution in ET compared to CN within the 1C fertigation system, P accumulation was notably higher in CN than in ET. This observation might be attributed to the fact that root analysis was not conducted; therefore, it remains plausible that any excess P is encased in the roots. As indicated by the accumulation data, P content within the plant tissues did not exhibit a decline following the cessation of nutrient supplementation. This phenomenon suggests a potential release of P from vacuoles (Shiponi and Bernstein, 2021a; Shiponi and Bernstein, 2021b), serving as a defense mechanism against P toxicity in the shoot (Hawkesford et al., 2012). This mechanism prevents the exposure of shoot cells to damaging P concentrations by compartmentalizing excessive P supply. The compartmentalization of P is recognized as a fundamental mechanism for averting the accumulation of cytoplasmatic P to toxic levels. Under adequate P nutrition, approximately 70–95% of intercellular phosphate (Pi) is sequestered within vacuoles. Consequently, the regulation of transporters under varying P conditions facilitates the maintenance of Pi cellular homeostasis (Liu et al., 2016). So far, there is no information about P transporters in *Cannabis sativa* L. This information is needed to understand better P remobilization and translocation in the cannabis plant (Shiponi and Bernstein, 2021b). An alternative explanation for this unexpected result could be a potential nutrient lockout effect in the ET due to the elevated P concentration in the solution. Phosphorus has an inclination to form complexes with soil minerals, such as iron Fe, potentially diminishing its overall bioavailability. However, it is essential to note that such an effect was observed by Mardamootoo et al. (2021) exclusively in soil-based cultivation systems. When soluble phosphatic fertilizers are applied to soils, they initially dissolve, causing an immediate increase in soil solution P concentration. Subsequently, P primarily engages in adsorption and precipitation processes (Prasad and Power, 1997). The reactions that transpire among phosphate ions in the soil solution, soil constituents, and non-phosphatic components in the fertilizers primarily sequester P from the solution phase, rendering phosphate less soluble over time (Sample et al., 2015). This phenomenon is commonly referred to as P fixation, adsorption, or retention. Consequently, P becomes markedly immobile in soils and tends to remain in proximity to the point of application (Prasad and Power, 1997; Mardamootoo et al., 2021). In plant cells, P serves as a critical component of nucleic acids, membrane lipids, and phosphorylated intermediates involved in energy metabolism. As a result, maintaining cellular phosphorus homeostasis is essential for ensuring the normal function of various physiological and biochemical processes (Raghothama, 2015). The elevated P concentration observed in the 2C system compared to the 1C system may likely be attributed to an increase and fluctuations in pH within the 1C regime, a phenomenon in line with the findings of Lefever et al. (2017). The higher pH levels, reaching up to 6.95 in the nutrient solution, could lead to the partial precipitation of phosphates by calcium (Ca^2+^) and magnesium (Mg^2+^). This precipitation could result in the formation of insoluble and consequently unavailable salts within the hydroponics solution (Lee et al., 2017).

K exhibits a clear accumulation advantage in the ET variant in 1C as a highly bioavailable plant element. In accordance with (Bernstein et al., 2019a), it is noteworthy that cannabis plant stems tend to exhibit a preference for K accumulation, as evidenced by concentrations similar to those found in fan leaves. Typically, this nutrient’s temporary storage occurs in the stem’s xylem or phloem parenchyma. The K concentration in cannabis stems remained notably high at the end of the developmental period, a phase characterized by high nutrient demand, indicating active accumulation. While our study did not measure sugar leaves but entire inflorescences, where K accumulation was slightly higher than in the stem, the results for K suggest that the nutrient concentration does not significantly decrease in tissues after nutrient cessation, indicating its excessive accumulation. This phenomenon, known as the luxury consumption of K and its temporary storage, has been observed in various plant species (Marschner and Marschner, 2012). It is pertinent to add that, based on the experiments conducted by Saloner et al. (2019) and Saloner and Bernstein (2022), there appears to be no competition for nutrient uptake between K and the uptake of N and P at suitable concentrations. Yep and Zheng (2021) studied the yield of *Cannabis sativa* inflorescence with K fertilizer in aquaponic solutions, similar to hydroponic systems. The added K and micronutrient fertilizers did not affect vegetative growth or leaf physiology. However, a positive linear correlation was observed between K concentration in the nutrient solution and both apical inflorescence yield (g/plant) and total inflorescence yield. Additionally, K fertilizer enhanced the harvest index.

Similarly, Fe demonstrates higher accumulation in the ET in 1C, except for the end of the cultivation, where the trend reverses. In contrast, interestingly, within the 2C fertigation system, significant differences in element accumulation between CN and ET treatments for any of the elements were absent. Generally, the differences in nutrient accumulation were predominantly insignificant when comparing CN and ET. These distinctions can be considered negligible, except for a few isolated instances. Furthermore, the disparities between the 1C and 2C regimens can be attributed to the 2C system’s superior control over nutrient solutions, ensuring stable and precise nutrient concentrations (Malík et al., 2023). Analyzing these variations in nutrient accumulation between the 1C and 2C fertigation systems provides valuable insights into how fertigation systems shape the ‘ionome’ composition of cannabis plants. We found that N concentrations favored the 2C system during later flowering stages, notably in weeks 8 through 11. P concentrations in flowers also showed advantages for the 2C system, particularly in weeks 7, 9, and 10. Conversely, P concentrations in leaves favored the 1C regimen, especially in weeks 9 through 12. K concentrations in leaves followed a similar trend, favoring the 1C system in weeks 6, 7, and 9. Fe concentrations exhibited variations favoring both systems, with Fe in flowers favoring the 1C system at week 8, whereas Fe in leaves and stems favored the 1C system in weeks 11, 12, and 6, respectively. These findings underscore the complexity of nutrient dynamics in fertigation, with the 2C regimen’s precise control over nutrient concentrations offering valuable insights for optimizing nutrient uptake and enhancing plant development. Considering the results regarding all concentrations of the studied elements in all the plant organs examined, our third hypothesis concerning the influence of the changed nutritional regime on the composition of the ‘ionome’ was confirmed.

Regarding the dry weight of above-ground organs, the ET had minimal effects compared to the CN. When comparing CN with ET in the 1C fertigation system, statistically significant changes were primarily observed in the dry weight of leaves. The supplementation of P did not induce any significant alterations in cannabinoid concentration within the flowers, but it led to a decrease in concentration within the inflorescence leaves. It is noteworthy that studies conducted with industrial hemp have demonstrated varying responses to P fertilization depending on growing conditions and cultivars (Bernstein et al., 2019b). In instances where the initial P concentration in the soil is relatively high, the additional P application tends to have limited effects. Phosphorus is indeed a critical nutrient required in relatively substantial amounts by plants. However, conventional practices in the medicinal cannabis industry involve the application of higher P concentrations than those typically used for most other crops. This practice is rooted in the belief that cannabis plants necessitate elevated P concentrations to optimize their functionality and enhance yield (Bevan et al., 2021). It is worth noting that mineral nutrition significantly influences plant morpho-development, and our findings indicate that supplemental P beyond optimal requirements does not contribute to further increases in plant biomass. This outcome aligns with prior research on hemp (Vera et al., 2010). It reinforces our conclusion that medicinal cannabis exhibits a broad optimal P-level range (Shiponi and Bernstein, 2021a). Interestingly, in the ET, plants exhibited greater leaf biomass during the early generative phase of flowering. However, their leaf biomass declined as flower maturation progressed and became statistically more minuscule than the CN. These findings intriguingly suggest that ET group plants in the 1C system displayed a more foliated profile at the onset of flowering, a phenomenon in line with the work of Hawkesford et al. (2012). Shiponi and Bernstein (2021a) recommend a minimum P requirement of 15 mg·L^−1^ P, with a suggested application rate of 30 mg·L^−1^, based on the functional physiology and ionome profiling revealing genotypic variability in P sensitivity. Notably, our tested P application rates, ranging from a minimum of 32 mg·L^−1^ to a maximum of 93 mg·L^−1^, significantly exceed those employed in the reference study. Nevertheless, fewer leaves were present in the inflorescence during the last two weeks of cultivation in the 1C system. This phenomenon could simplify post-harvest inflorescence arrangement and contribute to a higher quality of harvested inflorescence. In conclusion, when comparing CN and ET at 1C, it becomes evident that ET produces noticeably more biomass in flowers only at week 8, potentially suggesting this is an optimal harvesting time. However, in the subsequent weeks, flower biomass continued to increase. As we will explore in the following discussion, cannabinoid concentrations, mainly THC, were also found to be higher in the later stages of cultivation. Therefore, a later harvest time beyond week 8 seems preferable to optimize economic efficiency while maximizing yield. When comparing CN with ET in the 2C fertigation system, statistically significant differences were observed only during the 11th week of cultivation. Comparing the two fertigation systems, 1C and 2C, statistically significant differences in the dry weight of leaves and flowers favored 1C at weeks 6 and 7 and favored 1C only for flowers at week 10. However, no statistically significant differences were observed at the end of cultivation. In summary, the results of the dry weight yield of biomass suggest that neither the altered nutritional regime of the ET variant nor the type of fertigation system had a statistically conclusive effect on the final inflorescence harvest mass of medicinal cannabis. Therefore, our first hypothesis about the inflorescence yield was only partially confirmed because we recorded a statistically significant difference between CN and ET in the harvest period only at week 11 in the 2C system.

Cannabinoid levels represent a critical aspect in assessing the excellence of medicinal cannabis. Subjects purchasing the resulting cultivated product – the dry female inflorescence of medicinal cannabis – when selecting a supplier are oriented largely according to THC concentrations and, eventually, CBD in dry matter (Zhu et al., 2021). Since cannabinoids are the constituents conferring exceptional value to cannabis, it is imperative to conduct additional research into the influence of mineral nourishment on cannabis productivity and the association between yield and potency (Bevan et al., 2021). Prior research suggests that plants’ mineral nutrition can influence the production of secondary metabolites in cannabis (Caplan et al., 2017; Saloner and Bernstein, 2021; Saloner and Bernstein, 2022). An inverse correlation between cannabis yield and cannabinoid concentrations has been observed in previous studies. These studies have consistently reported a linear decrease in cannabinoid concentrations with increasing yield (the dilution effect) (Caplan et al., 2017; Yep et al., 2020; Shiponi and Bernstein, 2021a). This effect was not observed to a significant extent in our case. Additionally, future investigations should encompass the examination of other compounds acknowledged for their influence on product quality. Treatments involving metals such as Fe and Cu have demonstrated the potential to enhance secondary metabolite production in numerous plant species (Gorelick and Bernstein, 2014). The impact of metals, including Ni, Ag, Fe, and Co, on bioactive compound production is attributed to their ability to modulate various aspects of secondary metabolism Zhao et al., 2001). In the context of hemp cultivation, it has been observed that N supplementation leads to increased plant height and biomass (Papastylianou et al., 2018). Conversely, it is noteworthy that P or K fertigation treatments have shown limited efficacy in eliciting substantial responses (Aubin et al., 2015). It is important to acknowledge that while these findings provide valuable insights into plant growth dynamics, their direct relevance to cannabis cultivation is somewhat limited. In the context of cannabis, the paramount consideration is the concentration of therapeutic cannabinoids, a factor that far outweighs concerns related to overall biomass.

This study correspondingly delved into the intricate relationship between fertigation systems and cannabinoid concentrations in medicinal cannabis, providing insights contributing to the growing body of knowledge in this field. Our findings elucidate critical factors influencing both yield and cannabinoid potency and yield.

These findings, confirming our 4th hypothesis, align with prior research, particularly studies exploring the impact of fertigation systems on cannabis cultivation. For example, a study comparing recirculation and drain-to-waste systems found that recirculation led to higher yields of THCA and CBNA, the prominent cannabinoids in medical cannabis chemotype I, but resulted in lower sesquiterpene concentrations. Drain-to-waste, however, allowed for better control over nutrient delivery but consumed more resources and yielded fewer monoterpenes and THCA (Malík et al., 2023). In our case, there was also a significant increase in THC yield in the recirculation system (up to 182% more in the 10th week).

A previous investigation (Bernstein et al., 2019b) noted that employing mild nutritional treatments, which closely adhered to the optimal range for plant growth, resulted in subtle changes in plant development. However, there emerged an observable influence on the cannabinoid profile. These findings suggest the intriguing possibility that slight adjustments in nutritional status could play a role in modulating secondary metabolism in cannabis. This statement is also confirmed by our results, where the combination of changes in the yield and concentrations of the dominant cannabinoid (THC) resulted in an increase in the yield of total THC (g·m^2^) by an average of 48% during harvest period for the variant with enriched nutrition.

In accordance with prior research, our findings align with established knowledge, indicating that the lowest recommended P supply for optimal yield is 30 mg·L^−1^, and optimal yields are maintained at concentrations up to 90 mg·L^−1^ (Shiponi and Bernstein, 2021b). An earlier study also observed that the impact of increased P supplementation on medicinal cannabis is dependent upon the specific organ and compound. Specifically, the concentrations of key cannabinoids such as THC, CBD, CBN, and CBG remained unaffected by the treatment of enhanced P supplementation. The addition of P did not significantly affect cannabinoid concentrations within the flowers; however, it did lead to a reduction in cannabinoid concentration in the leaves of the inflorescence (Bernstein et al., 2019b). It is worth noting that in our study, the experimental ET variants were exposed to elevated P levels, reaching as high as 93 mg·L^−1^ P toward the end of cultivation. Importantly, similar to existing research, we did not observe any significant impact of elevated P levels in the nutrient solution on the production of cannabinoids. In contrast, interestingly, an older study from Coffman and Gentner (1977) reported a notable increase in total cannabinoid content per plant with higher P supply. This is consistent with our finding that the nutrient-enhanced variant achieved higher THC yields.

In line with previous research, our findings confirm that increasing K supply beyond 60 mg·L^−1^ K does not yield beneficial effects. It is noteworthy that variations in K supply had a relatively modest impact on both cannabinoid and terpenoid levels within the plant (Saloner and Bernstein, 2022). Notably, the decrease observed in these compounds was generally mild and less pronounced compared to the significant influence of N on secondary metabolism (Saloner and Bernstein, 2021). It is worth noting that in our study, the ET variant had elevated K levels up to 265 mg·L^−1^. Similarly, we did not observe any significant impact of increased K levels on the augmentation of cannabinoid concentrations. However, it is necessary to point out again that there was a significant increase in THC yield per square meter in ET variant.

To the best of our knowledge, no prior studies have explored the influence of Fe concentration in the nutrient solution on the content of secondary metabolites in the harvested inflorescence of medicinal cannabis. Our investigation revealed a notable outcome, which examined the effects of elevated macro- and micronutrient levels, including iron, in the ET variant. Contrary to some expectations, the increased iron content did not substantially impact the final cannabinoid content of the harvested medicinal cannabis inflorescence.

Exploring additional variables, such as specific nutrient formulations and variations in environmental conditions, will be essential to comprehensively understand their influence on cannabinoid production in indoor-cultivated cannabis plants.

The complexity of the interactions between various nutrients, environmental factors, and cannabinoid biosynthesis remains a rich area for investigation. Refining our understanding of these dynamics can unlock even more precise methods for optimizing cannabinoid production in indoor-cultivated cannabis. Such advancements hold great promise for both enhancing the therapeutic potential of indoor-cultivated cannabis products and improving their overall cultivation efficiency. This knowledge is valuable for cultivators seeking to maximize both yield and cannabinoid potency in a sustainable and resource-efficient manner.

## 5 Conclusion

In this study, we explored the intricate relationship between enhanced nutrition, different fertigation systems, and the ionome composition, biomass, and cannabinoid content of indoor-cultivated cannabis plants, comparing the yield of THC in the harvested inflorescence per square meter. Our research reveals multifaceted connections between nutrient supply, plant development, and valuable secondary metabolites production in medicinal cannabis. Significant nutrient variations, including N, P, K, and Fe concentrations in various plant organs under the augmented nutritional regime, result from shifts in nutrient uptake, translocation, and allocation due to changes in nutrient supply. This underscores the pivotal role of nutrition in shaping plant development, functionality, and metabolic processes, aligning with prior studies. Our findings emphasize the need for precise nutrient management strategies in cannabis cultivation. Adjusting nutrient regimens in different fertigation systems yielded significant outcomes. Notably, increased iron content in the ET variant did not substantially impact cannabinoid content, challenging assumptions. The impact of different fertigation systems on nutrient concentrations within plant tissues was evident. Adjusting the nutrient regimen in the 2C system led to a significant 108% increase in P accumulation in the stems in the 11th week, and there was a preference for K accumulation in cannabis stems, with up to a 77% increase in K levels in the ET. The ET increased the Fe concentrations, mainly in stems, up to 189%. The yield of THC in flowers per square meter with enriched nutrition increased by up to 50.7% compared to the control variant. There was even an 182% increase in THC yield in the recirculation system in the case of the ET comparison. As we conclude this study, we invite further investigations into the nuanced relationship between mineral nutrition and cannabis productivity, considering the complex interplay between yield and potency. Our research resonates beyond the scientific realm, offering actionable guidance for cultivators striving to maximize both yield and cannabinoid potency. While our study has yielded valuable insights into the relationship between fertigation systems and cannabinoid concentrations in indoor-cultivated cannabis, it is essential to acknowledge that further investigations should specifically delve deeper into the impact of enhanced nutritional compositions within the nutrient solution.

## Data availability statement

The raw data supporting the conclusions of this article will be made available by the authors, without undue reservation.

## Author contributions

JV: Conceptualization, Data curation, Formal analysis, Investigation, Methodology, Validation, Visualization, Writing – review & editing. MM: Conceptualization, Data curation, Formal analysis, Investigation, Methodology, Validation, Writing – review & editing. JŠ: Data curation, Formal analysis, Validation, Visualization, Writing – original draft. PT: Project administration, Resources, Supervision, Validation, Writing – review & editing.
