# Supplementary figures and images for: Effect of augmented nutrient composition and fertigation system on biomass yield and cannabinoid content of medicinal cannabis (Cannabis sativa L.) cultivation
Source: Front Plant Sci. 2024 Jan 24;15:1322824. doi: 10.3389/fpls.2024.1322824 (PMC10847352; doi:10.3389/fpls.2024.1322824)

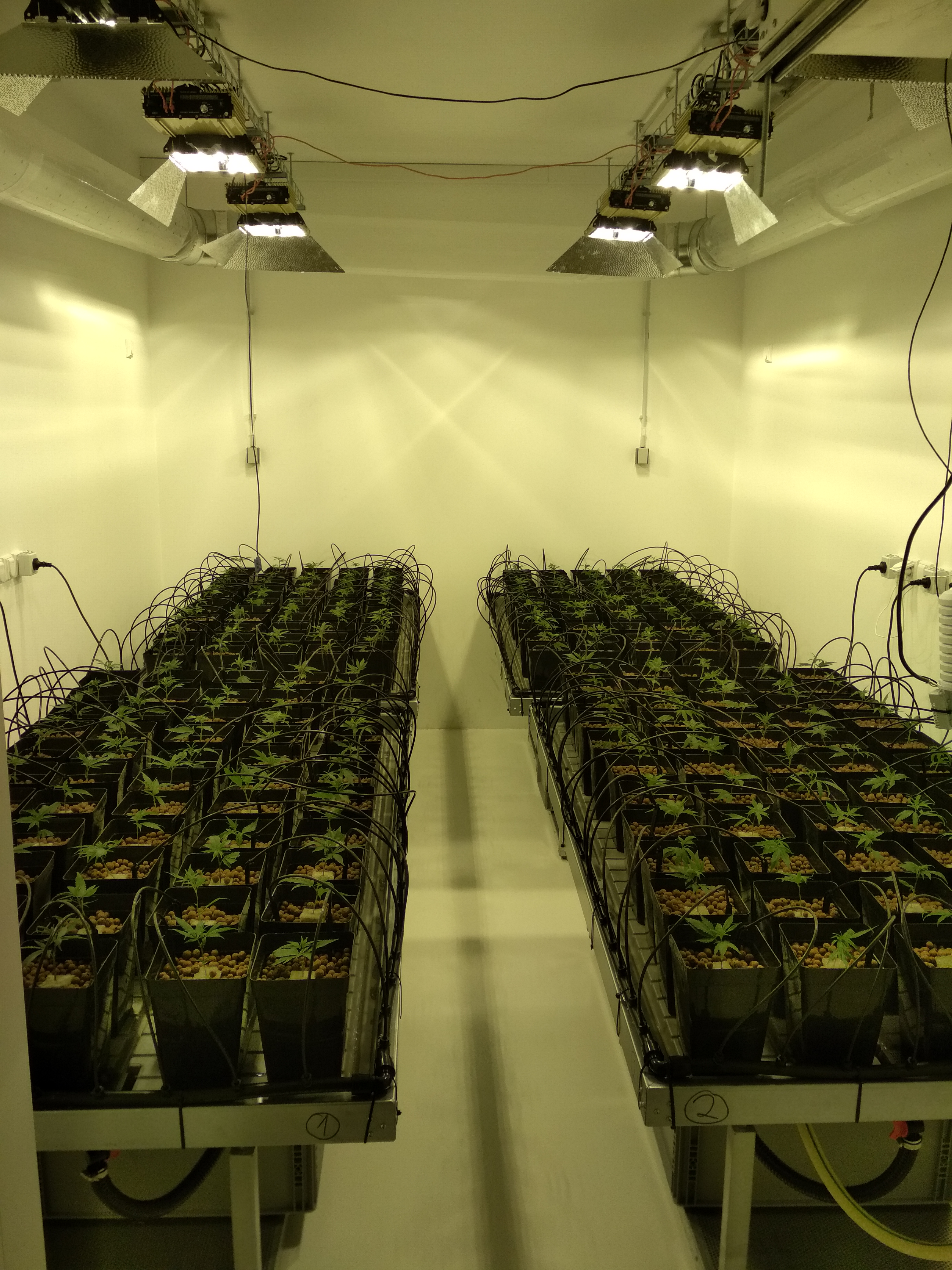

Supplement: Supplementary file 1 [file DataSheet_1.zip › Fotky experimentu/Grow room 1.jpg]

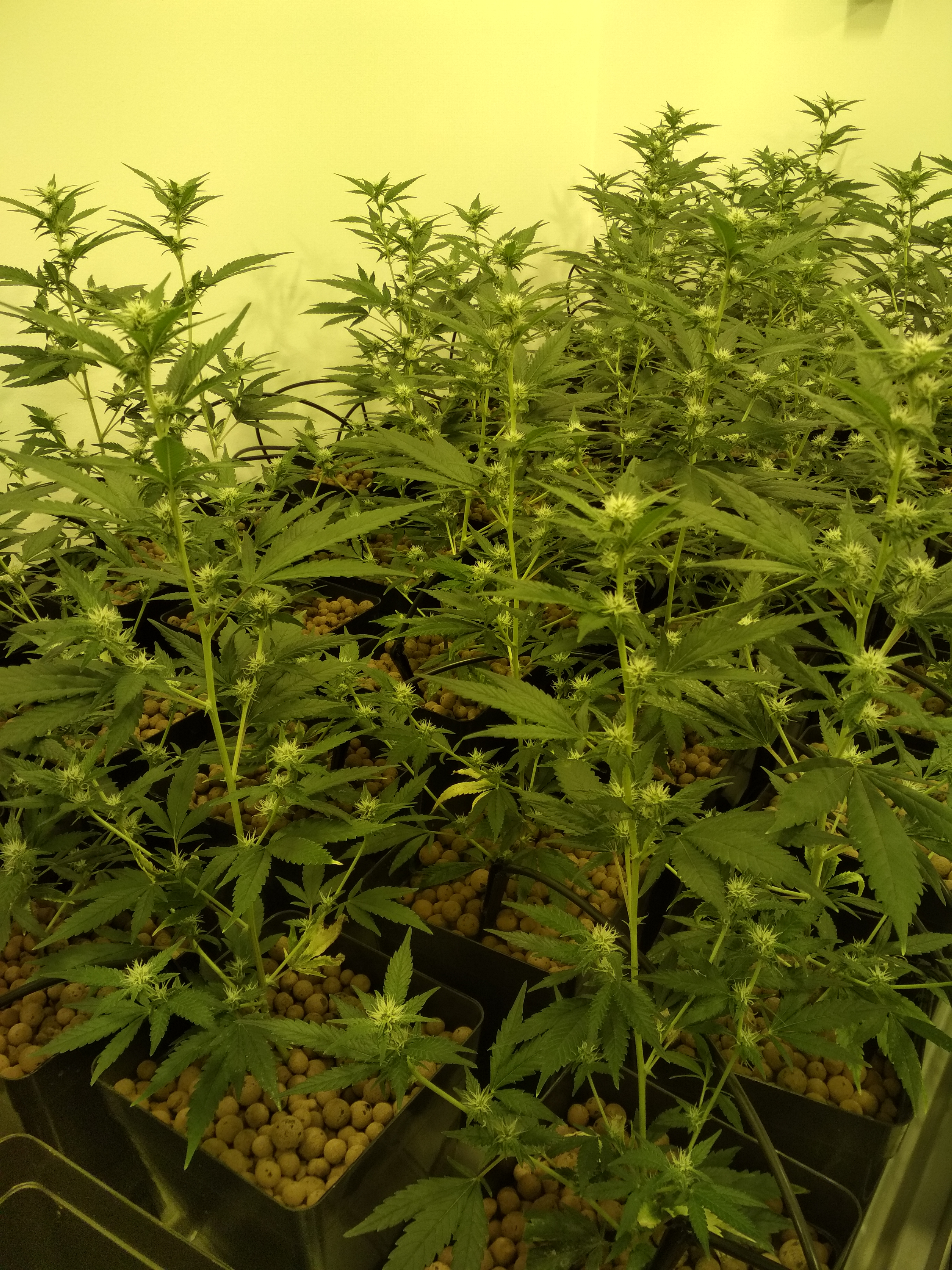

Supplement: Supplementary file 1 [file DataSheet_1.zip › Fotky experimentu/Grow room 2.jpg]

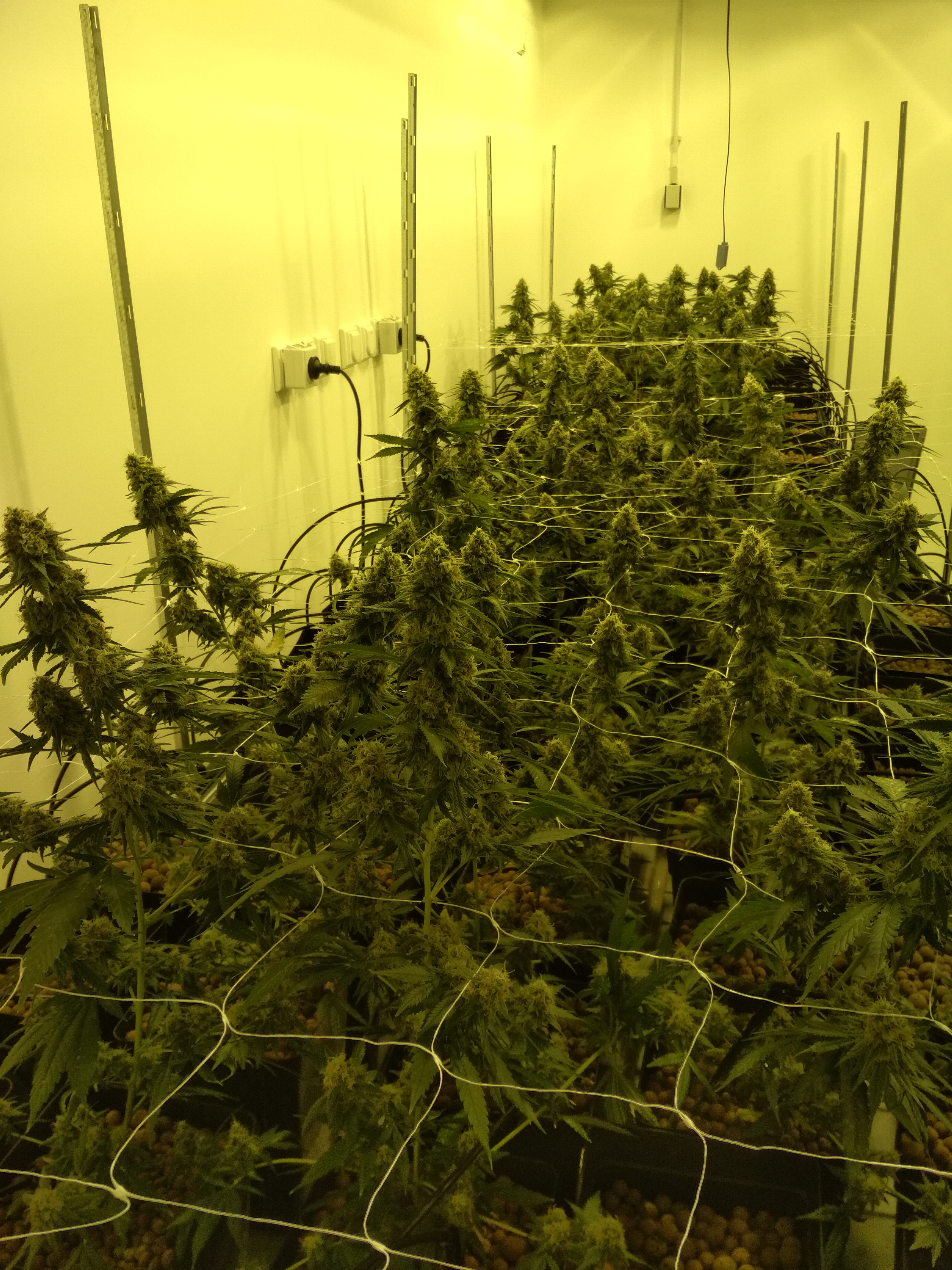

Supplement: Supplementary file 1 [file DataSheet_1.zip › Fotky experimentu/Grow room 3.jpg]

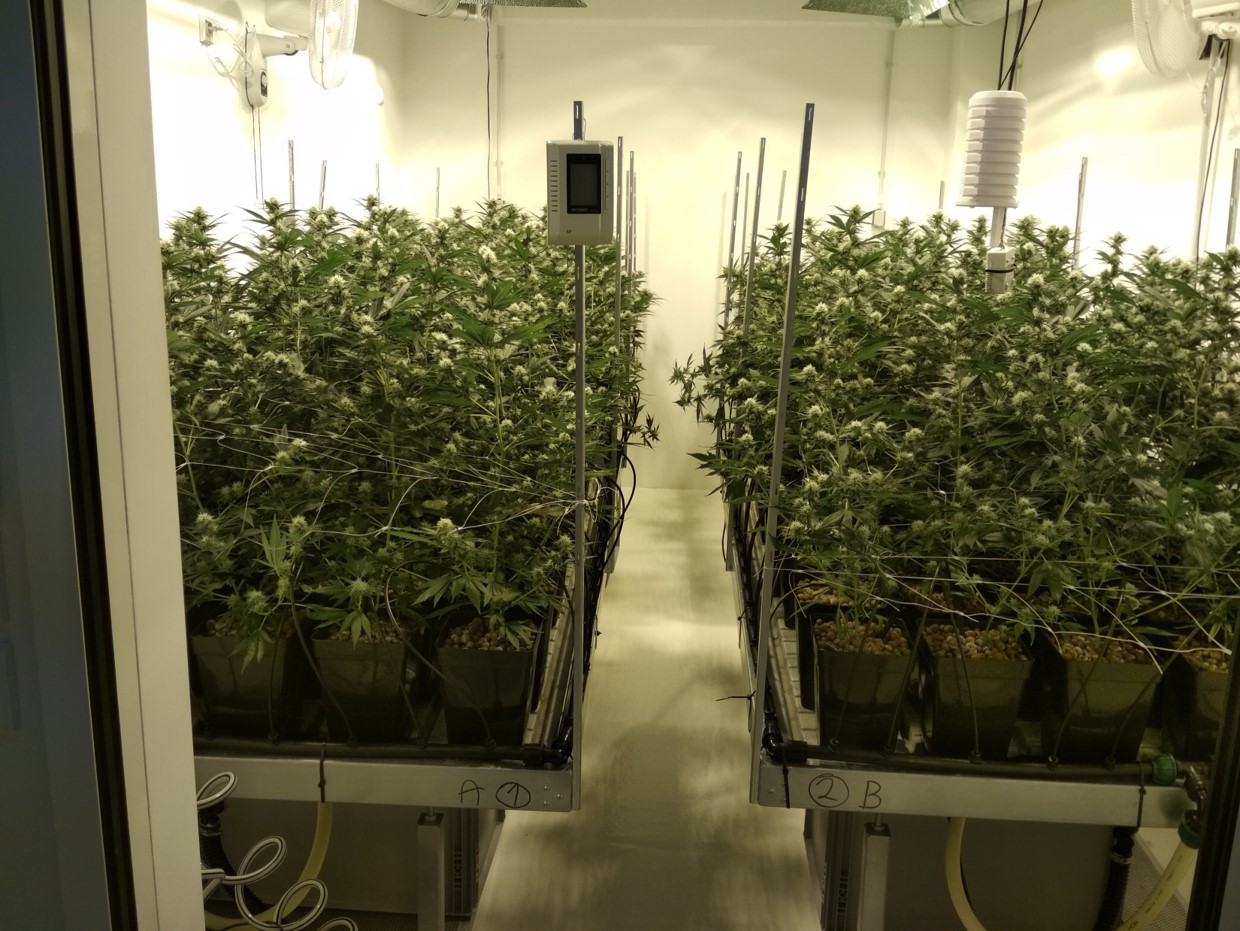

Supplement: Supplementary file 1 [file DataSheet_1.zip › Fotky experimentu/Grow room 4.jpg]

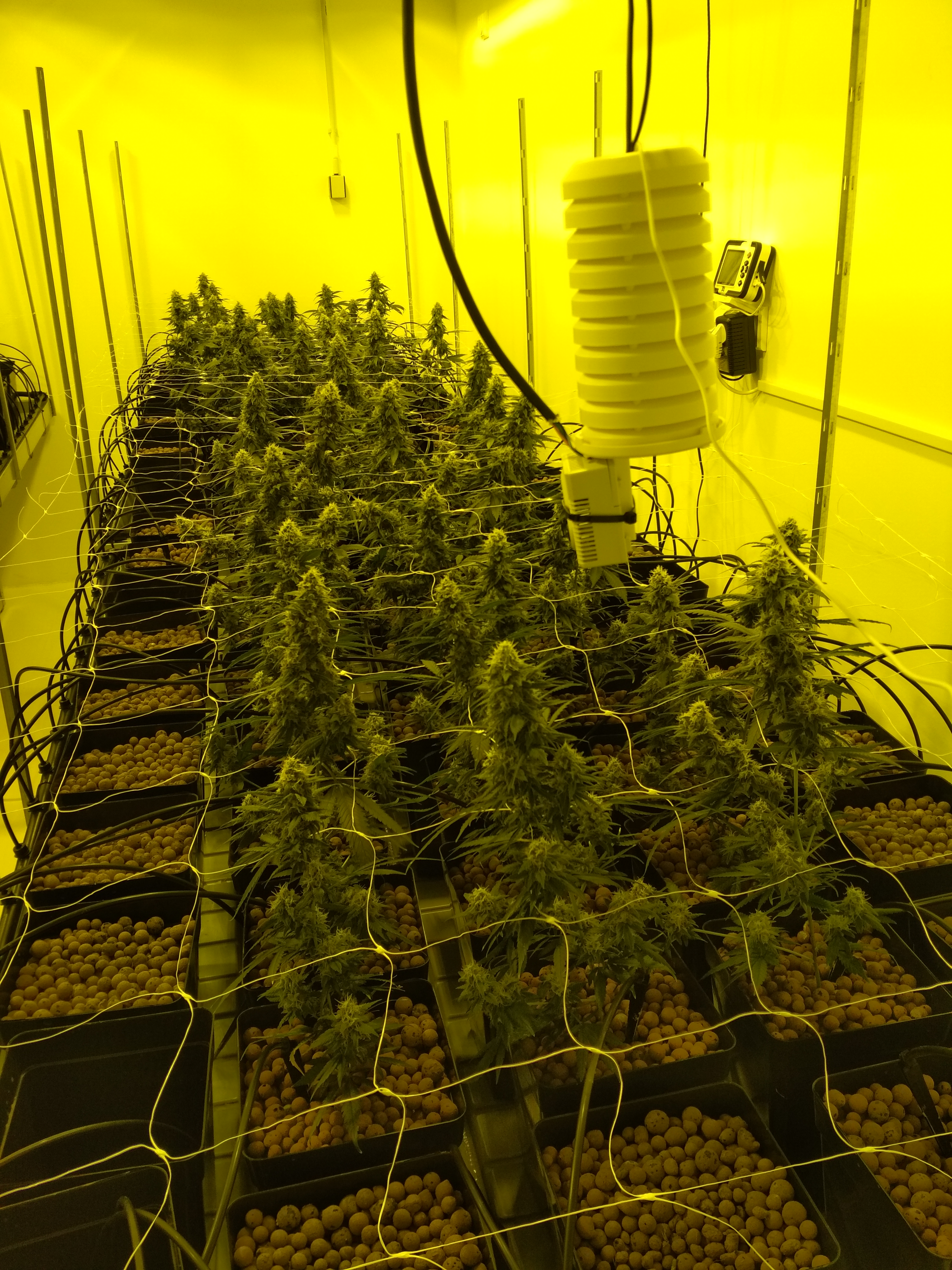

Supplement: Supplementary file 1 [file DataSheet_1.zip › Fotky experimentu/Grow room 5.jpg]
